# Treatment Strategies for Extramammary Paget Disease: A Narrative Review of Current Status and Future Directions

**DOI:** 10.3390/cancers18162587

**Published:** 2026-08-11

**Authors:** Azusa Miyashita, Satoshi Fukushima

**Affiliations:** Department of Dermatology and Plastic Surgery, Faculty of Life Sciences, Kumamoto University, Honjo 1-1-1, Chuo-ku, Kumamoto 860-8556, Japan; s_fukushima@kumamoto-u.ac.jp

**Keywords:** extramammary paget disease, surgical treatment, non-surgical treatment, radiation therapy, chemotherapies, immune checkpoint therapy, anti-HER2 antibody therapy

## Abstract

Background: Extramammary Paget disease (EMPD) is a rare epithelial malignancy that arises in the apocrine gland-bearing skin. Although localized intraepidermal EMPD generally follows an indolent clinical course, invasive EMPD is associated with lymph node metastasis, distant dissemination, and poor survival. Owing to its rarity, high-level evidence is limited and treatment strategies remain incompletely standardized, particularly for advanced disease. Recent advances in molecular profiling have revealed actionable therapeutic targets and created new opportunities for precision medicine. Methods: A narrative review was conducted using the PubMed database through May 2026. Relevant literature regarding the epidemiology, prognostic factors, surgical and non-surgical management, lymph node management, radiotherapy, systemic therapies, molecularly targeted therapies, and future therapeutic directions for EMPD is reviewed and summarized. Results: Surgical excision remains the standard treatment for resectable localized EMPD. Margin-controlled approaches, including Mohs micrographic surgery, complete circumferential peripheral and deep margin assessments, and mapping biopsy-guided excision, have contributed to improved local disease control. Radiotherapy, topical imiquimod, and photodynamic therapy provide alternative treatment options for patients who are unsuitable candidates for surgery; however, recurrence remains a major challenge. The management of regional lymph node metastases includes sentinel lymph node biopsy, lymph node dissection, and adjuvant radiotherapy in selected patients. For metastatic disease, conventional chemotherapy based on taxanes and fluoropyrimidine/platinum combinations has demonstrated moderate antitumor activity but limited durability. Molecular characterization of EMPD has identified several promising therapeutic targets. HER2 overexpression or amplification, observed in approximately 30–40% of cases, has emerged as the most clinically validated biomarker, with trastuzumab-based therapies demonstrating substantial efficacy. Immune checkpoint inhibitors have shown activity in selected patients, particularly those with a high tumor mutational burden, although predictive biomarkers remain inadequately defined. Additional emerging targets include androgen receptor signaling, TROP2, NECTIN4, FOXM1, and PIK3CA-associated pathways. Conclusions: In addition to conventional surgery- and chemotherapy-based approaches, biomarker-driven precision oncology is gaining attention as a treatment strategy for EMPD. HER2-targeted therapies, antibody–drug conjugates, and rationally selected immunotherapeutic strategies are expected to play increasingly important roles in the treatment of advanced disease. Continued translational research, international collaboration, and prospective clinical trials are essential to establish evidence-based treatment algorithms and improve outcomes in patients with this rare but potentially aggressive malignancy.

## Simple Summary

Extramammary Paget disease is a rare cutaneous adenocarcinoma, predominantly affecting older individuals and arising in apocrine gland-bearing areas, such as the vulva, scrotum, perianal region, and axilla. While most cases remain confined to the epidermis and follow an indolent course, a subset are invasive with regional lymph node involvement and distant metastasis, resulting in poor clinical outcomes. Surgical excision remains the standard treatment for localized disease. Management can be challenging in older patients. Radiotherapy, topical imiquimod therapy, and photodynamic therapy may provide alternative options for patients unsuitable for surgery; however, recurrence remains a concern. The optimal management of regional lymph node metastases remains to be established. Conventional chemotherapy has shown limited efficacy, and no standard global regimen has been established. Several promising therapeutic targets have been identified, including HER2, immune checkpoint pathways, androgen receptor signaling, TROP2, NECTIN4, FOXM1, and PIK3CA. HER2-targeted therapies, particularly antibody–drug conjugates, are emerging as promising approaches.

## 1. Introduction

Extramammary Paget disease (EMPD) is a rare skin cancer in which large, pale, atypical cells (Paget cells) proliferate within the epidermis. It is an epithelial malignancy that occurs in skin areas rich in apocrine glands, such as the vulva, perianal region, and axilla [1,2,3,4]. This differs from the clinical concept of mammary Paget disease, a subtype of breast cancer in which Paget cells originating from the ductal epithelium spread to the nipple or areola. Conditions that must be distinguished from EMPD include cancers of organs adjacent to the skin, such as rectal and anal canal cancers, urological cancers (e.g., urothelial carcinoma), and gynecological cancers, which have invaded the epithelium and reached the epidermis, presenting with findings characteristic of intraepithelial carcinoma, a phenomenon known as the Paget phenomenon [3,4].

**Figure 1:**
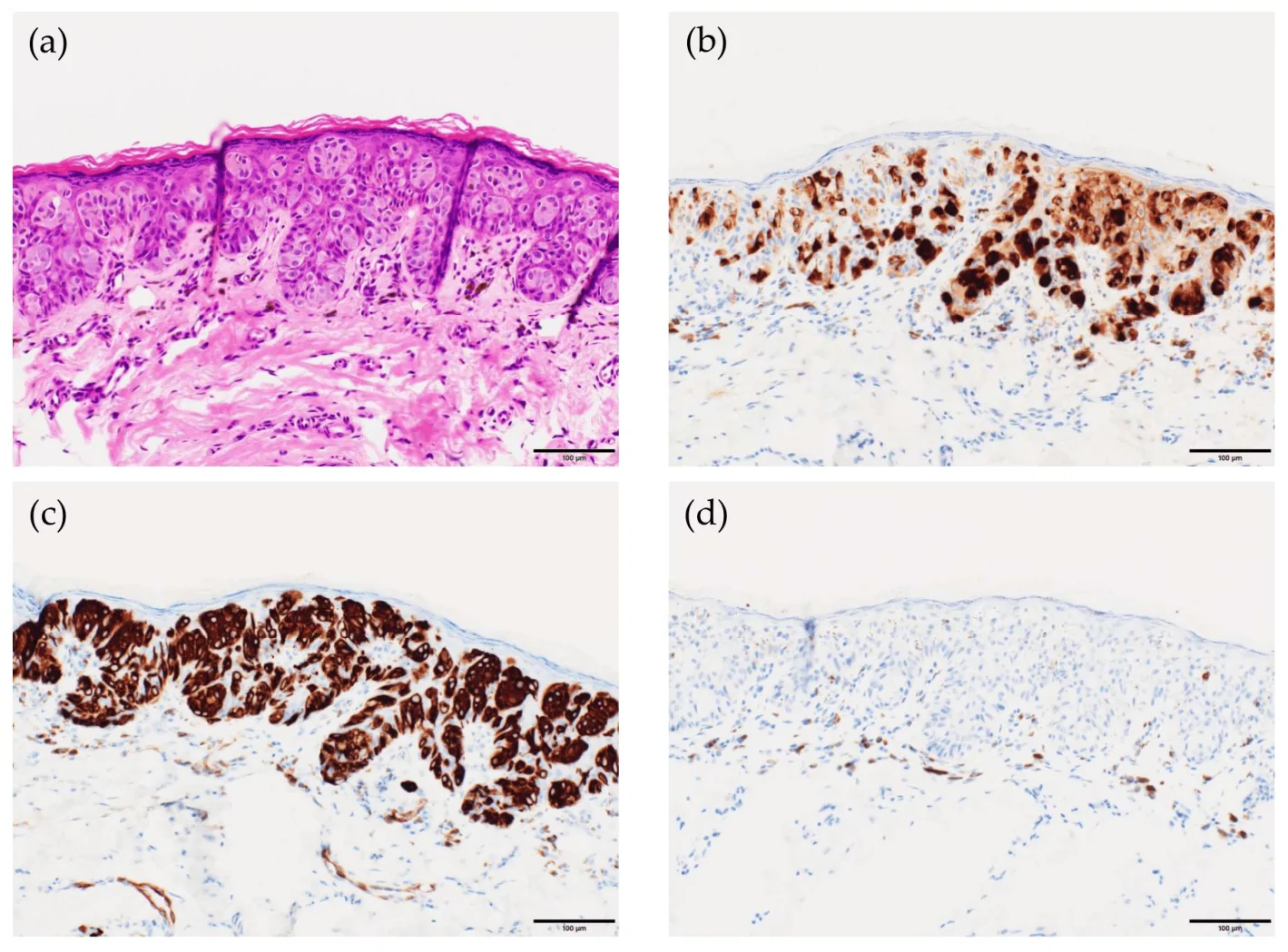
Typical examples of immunohistochemical staining patterns in primary EMPD. (**a**) Large, pale, atypical cells (Paget cells) are proliferating within the epidermis. (hematoxylin and eosin, ×200). (**b**) Positive immunostaining with GCDFP-15 is observed. (×200). (**c**) Positive immunostaining with CK7 is observed. (×200). (**d**) Negative immunostaining with CK20 is observed. (×200).

Certain immunohistochemical stains are useful for distinguishing between primary EMPD and secondary EMPD. Cytokeratin (CK)7 is a highly sensitive marker for EMPD, while CK20 has been reported to be positive in many cancers of the urinary tract and gastrointestinal tract [5,6]. Furthermore, gross cystic disease fluid protein (GCDFP)-15 has been reported to be strongly expressed in primary EMPD [5,6,7]. Primary EMPD typically exhibits a staining pattern of CK7(+), CK20(−), and GCDFP-15(+) (Figure 1), whereas secondary EMPD typically exhibits a staining pattern of CK7(+ or −), CK20(+), and GCDFP-15(−). Therefore, CK7, CK20, and GCDFP-15 can be used to help distinguish between primary and secondary EMPD. However, it is important to note that there are cases that do not fit this staining pattern [2].

EMPD primarily affects adults in their 60s and 70s [2,8]. The incidence rate of EMPD is higher in Asians than in Caucasians (0.28 vs. 0.13 per 100,000 population/year) [9,10]. The prevalence of EMPD regarding gender differs between Western and Asian populations. In studies on Caucasian populations, EMPD has a female predominance, with male-to-female ratios ranging from 1:2 to 1:7 [2]. In contrast, among East Asians, it has been reported that the male-to-female ratio tends to be higher for males, ranging from 1.5 to 3.37:1 [9,11,12,13].

EMPD generally presents as erythema, but it may also manifest as hypopigmented patches or erythematous patches with indistinct borders and may be accompanied by erosion or itching [2,8]. As the disease progresses, tumor cells invade the dermis and form nodules within erythematous patches. Since multiple lesions may occur discontinuously within a single anatomical region, it is necessary to carefully assess the extent of these lesions.

Carcinoma in situ, in which the tumor remains confined to the epidermis, generally follows a slow course and has a high survival rate [2,8]. Intraepithelial carcinoma generally progresses slowly and has a high survival rate [2,8]. It has been reported that 70% of EMPD cases are intraepithelial carcinoma and 30% are invasive carcinoma; in cases of invasive carcinoma, the risk of metastasis is high and the prognosis is poor [14]. Distant metastasis has been reported in 10–20% of patients with EMPD [15], and 50% of these patients die within 1 year [14], suggesting a high degree of malignancy. The major factors associated with poor prognosis include tumor thickness (depth of invasion), lymphatic invasion, number of lymph node metastases, and presence or absence of distant metastases [2,6,8].

When curative resection is feasible, the standard treatment is surgical resection. To reduce the recurrence rate, wide local excision (with margins of 1–5 cm) and Mohs micrographic surgery (MMS) are employed [1,2,8]. In contrast, for cases where surgery is difficult due to advanced age or comorbidities or for recurrent cases, non-surgical treatments such as topical imiquimod, radiation therapy, and photodynamic therapy (PDT) are alternative options [1,2,8]. Conventional chemotherapy regimens based on docetaxel (DOC), 5-fluorouracil (5-FU), and cisplatin have been used for metastatic EMPD; however, their efficacy is limited, and no standard treatment regimen has been established [2,8,9]. In recent years, new therapeutic approaches, such as anti-human epidermal growth factor receptor 2 (HER2) antibody therapy targeting HER2 overexpression, which is observed in 30–40% of cases, as well as hormone therapy targeting the androgen receptor and immune checkpoint inhibitors, have garnered significant attention [2,8,16]. There are clinical guidelines on the treatment of EMPD reported from Europe and the United States, and Japan [3,4]. Several discrepancies regarding treatment strategies were observed in these two clinical practice guidelines. Discrepancies were noted in the methods for determining surgical margins for the primary tumor, the level of recommendation for sentinel lymph node biopsy, and treatment strategies for lymph node metastases.

Based on recent research findings, this review provides a comprehensive analysis of the latest epidemiological data on EMPD, clinical and pathological factors influencing prognosis, and treatment modalities, ranging from surgical interventions to the latest systemic therapies. It also presents the current challenges and future prospects in the management of this disease.

## 2. Materials and Methods

We conducted a literature review using PubMed up to May 2026 using the following keywords: extramammary Paget disease (EMPD), surgical treatment, non-surgical treatment, radiation therapy, systemic treatment, immunotherapy, chemotherapy, targeted therapy, and guidelines. This article does not address Paget disease of the breast or the Paget phenomenon.

This is a narrative/descriptive review. Studies were selected based on relevance, and no formal Preferred Reporting Items for Systematic Reviews and Meta-Analyses methodology, risk-of-bias assessment, or meta-analysis was conducted.

## 3. Results

### 3.1. Treatment for Primary Lesions

Radical surgical resection is the standard treatment for primary resectable EMPD. Currently, the approach to lateral resection margins varies depending on different schools of thought and available techniques. In cases where radical surgery is difficult due to poor general health, treatment options include radiation therapy, topical imiquimod, and PDT; however, it is important to note that these options must be selected based on the regulatory approval status in each country.

#### 3.1.1. Surgical Treatment

Surgical treatment is the primary approach when curative resection is feasible. Generally, when there is a low likelihood of clinically evident lymph node metastasis and the primary tumor can be resected with adequate margins, wide excision of the primary tumor is considered the first-line curative treatment. Since EMPD commonly occurs in the vulva and perianal region, a multidisciplinary approach involving collaboration among dermatology, gastrointestinal surgery, urology, and gynecology is necessary to achieve curative resection. Because EMPD lesions tend to spread in an unclear and multifocal manner beyond grossly visible margins (subclinical extension), it is difficult to determine the appropriate extent of resection [17,18,19].

Clinical practice guidelines reported in Europe and the United States in 2022 (Evidence-Based Clinical Practice Guidelines for Extramammary Paget Disease) present a systematic review of treatment outcomes for wide local excision, with a mean surgical margin of 1.9 cm (standard deviation 1.0 cm) using MMS and complete circumferential peripheral and deep margin assessment (CCPDMA). Given the low recurrence and mortality rates associated with MMS and CCPDMA, these guidelines recommend excision via MMS or CCPDMA for both in situ and invasive lesions [4]. The lateral resection margins required to limit the residual tumor to 5% were 4 cm for lesions in the scrotum, penis, or vulva, and 3.5 cm for lesions in the perianal or axillary regions. However, only a few facilities are capable of performing these procedures. Clinical guidelines published in Japan in 2025 suggested performing a mapping biopsy when performing radical resection of the primary tumor. In routine clinical practice in Japan, mapping biopsies are performed around the lesion prior to wide excision [3]. Mapping biopsy is a method used to ensure negative surgical margins. It involves performing punch biopsies from the macroscopically visible margins of the lesion before radical resection and setting lateral margins that include biopsy sites where the absence of tumor cells has been confirmed. This procedure is minimally invasive. Regarding local recurrence rates, four case series reports indicated that the rates ranged from 1.9% to 11.5% in the group where lateral margins were determined by mapping biopsy and from 16.7% to 57.1% in the group where lateral margins were determined by macroscopic findings, showing a tendency for lower rates in the group that underwent mapping biopsy [20,21,22,23]. Regarding the setting of the lateral margins, there was variation among reports, ranging from 1 cm to 3 cm. One characteristic of EMPD is that the macroscopic margins of the lesion are often indistinct; in some cases, the tumor may extend beyond these margins or lesions may occur in a multicentric or ectopic manner. Therefore, there is a view that it is useful to determine lateral margins based on findings from mapping biopsy. Although no reports have examined deep margins in detail, since EMPD involves the spread of tumor cells along accessory structures such as the hair follicle epithelium, it is desirable to establish deep margins that take these characteristics into account.

Regarding following treatment after an incomplete resection (positive or close margins), the general principle is to perform an additional resection if possible; however, if re-resection is difficult from a functional or cosmetic standpoint, adjuvant radiation therapy is considered one of the treatment options. Japanese clinical guidelines classify radiation therapy as “one of the options” based on the results of a case series study showing that, in cases with positive or close margins following resection of the primary tumor, radiation therapy (total dose of 40–61.2 Gy in 20–34 fractions) administered without additional resection achieved high rates of local control and recurrence-free survival [3,24,25]. According to reports by Hata et al. and Itonaga et al., the local recurrence rate (LRR) was 0% in both studies; in the report by Hata et al., the 5-year local recurrence-free survival rate was 97% and the 5-year overall survival rate was 71% [24,25]. A systematic review by Tagliaferri et al. also suggests that radiation therapy (total dose of 40–61.2 Gy in 20–34 fractions) following positive or close margins should be considered, citing the relatively low rate of local recurrence (33%) as the reason [26]. In two retrospective studies from China, the LRR among patients with positive surgical margins who underwent postoperative adjuvant radiation therapy following surgery (primarily MMS) was 0% in both studies [27,28]; a total dose of 50–60 Gy (in 30 fractions) is recommended [28]. Grade 3 or higher acute skin toxicity and chronic skin toxicity were observed in approximately 17% and 10% of patients, respectively [27,28]. On the other hand, to the best of my knowledge, there are no reports examining the efficacy of adjuvant systemic therapy following incomplete resection; therefore, I believe there is currently little evidence to support the use of adjuvant systemic therapy following incomplete resection.

#### 3.1.2. Non-Surgical Treatment

When there is no evidence of metastasis, surgery is the primary treatment; however, surgical resection of the primary tumor may be difficult owing to factors unrelated to EMPD, such as comorbidities or poor patient performance status, or for reasons related to preserving function. For cases in which surgery is not feasible, noninvasive and minimally invasive treatment options such as radiation therapy, topical chemotherapy, and PDT are available. Although imiquimod, 5-FU, and bleomycin are potential options for topical chemotherapy, this section focuses on imiquimod, which has been the subject of numerous studies.

Imiquimod acts as an agonist of Toll-like receptor 7 and is thought to promote the production of cytokines such as interferon-α, interleukin-12, and tumor necrosis factor-α, thereby enhancing innate and cellular immune responses against tumors [29,30,31,32]. Although no standard treatment regimen has been established, two prospective single-arm intervention trials (conducted in the Netherlands and Japan) involved the topical application of imiquimod three times a week for 16 weeks [33,34]. These prospective intervention trials focused on in situ cases and achieved high response rates, ranging from 82.6% to 100%; however, the recurrence rates were also high, ranging from 60% to 66.7% [33,34]. Fatigue, pain at the affected site, and headache were reported in approximately 70%, 70%, and 40% of patients, respectively [33]. Japanese clinical guidelines suggest topical imiquimod therapy (not covered by health insurance in Japan) for primary lesions in cases of in situ cancer that are amenable to curative resection, but for which surgical treatment is not feasible [3]. According to clinical guidelines in Europe and the United States, imiquimod may be considered for cases of in situ cancer with a high risk of surgical complications; however, owing to the high recurrence rate, close follow-up is required [4]. Therefore, while imiquimod has some value as a treatment option for patients who cannot undergo surgery or radiation therapy, it is important to note that insurance coverage for EMPD varies by country and region.

PDT is a treatment method in which a photosensitizer is taken up by tumor cells, and laser light is then applied to selectively destroy the tumor cells [35,36]. Although photosensitizers can be administered intravenously or topically, studies on EMPD have reported the efficacy of PDT using systemic administration of hematoporphyrin derivative (HpD) or topical application of 5-aminolevulinic acid (5-ALA) [36,37,38]. According to a recent systematic review, the overall complete response rate for PDT is approximately 50–60%; the complete response rates for specific drugs are reported to be approximately 58% for 5-ALA and approximately 89% for HpD [36]. On the other hand, the recurrence rate for PDT monotherapy is relatively high at approximately 33–40%, and there are limitations to its long-term efficacy [35,36,37]. PDT is generally a well-tolerated treatment; however, caution is required regarding local pruritus and erythema as well as photosensitivity (in cases of intravenous administration) [36,37,38,39]. According to Western guidelines, PDT may be considered for in situ cases where the risk of surgical complications is high; however, owing to the high recurrence rate, close follow-up is required [4].

#### 3.1.3. Radiation Therapy

Since patients with EMPD are often older at the time of diagnosis and the disease frequently occurs in the genital and perianal regions, which are critical for urinary and bowel function, radiation therapy may be selected as a treatment option when surgical therapy is not appropriate. Radiation therapy targeting the primary tumor is classified as curative or palliative, depending on the objective. Curative radiation therapy is performed with the aim of curing the disease in cases where surgery is not recommended or is not feasible. It is generally planned to administer a daily dose of 1.8 to 2.0 Gy once a day, 5 days a week, for a total dose of 60 to 70 Gy. Palliative radiation therapy is administered to alleviate symptoms and improve patients’ quality of life in cases of bleeding from locally advanced primary tumors as well as pain caused by regional lymph node, skin, and bone metastases. Standard treatment involves a single dose of 8 or 30 Gy delivered in 10 fractions.

Japanese clinical guidelines suggest curative-intent radiation therapy for primary tumors in cases where curative resection is technically feasible, but surgery cannot be performed [3]. Although there are no prospective interventional or observational studies comparing curative radiotherapy with surgical therapy, three retrospective observational studies (single-arm studies), one from Switzerland and two from Japan, reported that patients were treated with 36–70.2 Gy (median 60 Gy) X-rays or electron beams [40,41,42]. Although the irradiation methods used were inconsistent, response rates were 100%. The recurrence rate was 26.7% over an observation period of 6–144 months (median, 30 months). At the 5-year mark, the local control rate was 71%, the disease-free survival rate was 63%, and the overall survival rate was 78% [40]. Adverse events were observed in 73.3% of patients; however, these were primarily radiation dermatitis and were manageable with topical therapy [41,42]. Western clinical guidelines also state that radiotherapy for curative treatment may be indicated in cases where surgery is not recommended or is difficult to perform, and the average recurrence rate following curative radiation therapy has been reported to be 30% or higher [4]. They propose that the treatment field should extend 3.5 cm beyond the clinical margins of the tumor [4]. In a systematic review by Tagliaferri et al., radiation therapy was proposed as an alternative treatment for primary tumors involving both intraepidermal and dermal invasion when surgery is not an option [26]. This review reports that the complete response rate among patients who underwent curative-intent radiation therapy was 92.6%, and the local control rate at 5 years ranged from 20% to 71%; it also notes that radiation doses of 60 Gy or less increase the risk of local recurrence [26].

The most common acute adverse event is radiation dermatitis (erythema, desquamation, and erosion) at the irradiation site; however, most cases are grade 2 or lower, transient, and manageable [26,43,44]. In addition, mucositis, diarrhea, and hematological toxicity (such as leukopenia) have been reported, depending on the site of irradiation [26,43]. Major late adverse events include skin atrophy, hyperpigmentation, and asymptomatic telangiectasia within the irradiation field [26,43,44]. Grade 3 lymphedema or skin infection may rarely occur with extensive irradiation [44].

### 3.2. Treatment for Regional Lymph Node Metastases

EMPD progresses beyond the stage of proliferation within the epidermis to invade the dermis. As the disease advances, it may metastasize lymphatically, leading to regional lymph node metastasis and, in some cases, distant lymph node metastasis beyond the regional nodes. Treatment strategies for regional lymph node metastasis include regional lymph node dissection when curative resection is feasible and radiation therapy when curative resection is difficult for any reason. Furthermore, in cases where clinically evident lymph node metastasis is not detected, sentinel lymph node biopsy (SLNB) can be performed to determine the presence of clinically occult lymph node metastasis [45,46].

#### 3.2.1. Sentinel Lymph Node Biopsy

SLNB is a lymph node biopsy technique based on the concept that if no metastasis is found in the sentinel lymph node, the first node reached by lymph flow from the primary tumor, it is highly likely that no metastasis has spread to the regional lymph nodes beyond that point. This approach avoids unnecessary lymph node dissection and is a well-established procedure for the treatment of melanoma. In Japan, this method became covered by health insurance for EMPD in 2020. Japanese clinical guidelines suggest performing SLNB when intradermal invasion of the primary tumor is suspected [3]. Several prospective and retrospective observational studies from Japan have been reported [47,48]; while some studies found no significant difference in overall survival (OS) between sentinel lymph node-negative and sentinel lymph node-positive groups, others reported a significant prolongation of OS in the sentinel lymph node-negative group. Furthermore, a correlation between the number of regional lymph node metastases and prognosis has been reported. Yoshino et al. reported a decrease in 5-year survival rates when two or more nodes were positive, while Kurooka et al. reported a decrease when three or more nodes were positive [49]. SLNB may be useful in predicting prognosis by determining the presence and number of lymph node metastases. In Japan, SLNB is suggested in cases where dermal invasion is suspected. However, it is important to note that no randomized controlled trials (RCTs) have compared survival between groups that underwent SLNB and those that did not; therefore, it remains unclear whether SLNB improves prognosis. Western clinical guidelines do not recommend SLNB for EMPD, partly because the utility of identifying microscopic lymph node metastases remains unclear [4].

#### 3.2.2. Regional Lymph Node Dissection

When clinical or pathological metastasis is detected in the regional lymph nodes, lymph node dissection is generally performed to control the local lesions.

In recent years, it has been reported that the number of regional lymph node metastases is an important prognostic factor for EMPD. However, reports on this number vary. While some studies indicate that cases with two or more regional lymph node metastases have a significantly poorer prognosis than those with one or fewer metastases [15,47,50], other studies suggest that metastases limited to one node on each side do not necessarily indicate a poor prognosis [51]. Furthermore, while some reports indicate a poor prognosis in cases with three or more regional lymph node metastases [52,53], they do not specify whether these cases involve metastases on one or both sides. Given this situation, Japanese clinical guidelines state that regional lymph node dissection may be a practical and effective option when lymph node metastasis involves a single node [3]; however, the efficacy of lymph node dissection in cases suspected of having multiple regional lymph node metastases remains unclear. Clinical guidelines in Europe and the United States do not include any descriptions of regional lymph node dissection. As multiple treatment options are available, including radiation therapy and systemic chemotherapy, in addition to lymph node dissection, the appropriateness of this procedure must be carefully evaluated. In cases of lymph node metastasis involving multiple regions, lymph node dissection alone may have limitations; therefore, the implementation of multimodal therapy, including radiation therapy, should be considered to improve prognosis. A retrospective observational study from Japan reported that in patients with three or more lymph node metastases, the group that received postoperative adjuvant radiation therapy following lymph node dissection showed a significantly improved survival rate (5-year survival rate: 75% vs. 0%) compared to the group that did not receive such therapy [53]. Consequently, the Japanese clinical guidelines suggest postoperative radiation therapy when three or more regional lymph node metastases are detected [3]. The Japan Clinical Oncology Group has initiated an RCT comparing disease-free survival and OS in patients with multiple regional lymph node metastases on one side, comparing cases treated with regional lymph node dissection alone versus those treated with a combination of lymph node dissection and postoperative radiation therapy (jRCT 1030260246).

#### 3.2.3. Radiation Therapy

In cases involving multiple lymph node metastases or when lymph node dissection is not feasible due to complications or other factors, radiation therapy is an important treatment option that may contribute to improved local control and OS [26,43,54].

Radiation therapy for regional lymph node metastases is classified into curative and palliative treatments, depending on the objective. Curative radiation therapy is performed to achieve cure and generally requires a total dose of 60 Gy or more to achieve control [3]. Although there are several case series on the use of curative radiation therapy alone for regional lymph node metastases, few provide detailed information on the long-term prognosis [26,43,55]. Although the complete response rate among patients who have undergone curative radiotherapy is extremely high at 92.6%, cases of recurrence within the radiation field do occur; therefore, attention must be paid to technical precision and tumor radiation resistance. European and American clinical guidelines state that when surgery is not recommended or not feasible, radiotherapy aimed at curative treatment may be an appropriate option [4]. Palliative radiation therapy is administered to alleviate symptoms such as bleeding and pain caused by locally advanced regional lymph node metastases with the aim of improving patients’ quality of life. The standard treatment regimen involves short-term radiation therapy, typically ranging from a single dose of 8–30 Gy delivered in 10 fractions [3]. Adverse events included acute and late toxicities. Reported acute toxicities include radiation dermatitis (erythema and erosion), mucositis, diarrhea, cystitis, and transient hematological toxicity (such as leukopenia) [26,43]. They are often grade 2 or lower, and improve with appropriate management. Late toxicities include skin atrophy, hyperpigmentation, and telangiectasia in the irradiated field [26,43]. Radiotherapy is a useful treatment option within the EMPD treatment regimen, particularly in cases where surgery is difficult.

### 3.3. Treatment for Distant Metastases

As EMPD is rare, no RCTs have evaluated the efficacy and safety of systemic chemotherapy in patients with unresectable metastatic disease. Although the clinical guidelines in Europe, the United States, and Japan suggest the use of systemic chemotherapy in patients with unresectable metastatic disease, there is currently no standard chemotherapy regimen.

#### 3.3.1. Chemotherapies

Treatment strategies for advanced or metastatic EMPD are currently left to the discretion of individual medical institutions; however, treatment primarily involves DOC, low-dose 5-FU/cisplatin (FP), and combination therapies involving these agents [9].

There have been several reports on first-line treatment regimens. In a multicenter retrospective observational study, DOC monotherapy was found to be the most commonly used regimen in Japan and was adopted as the first-line treatment in approximately 66–69% of cases [9]. In a multicenter retrospective observational study conducted in South Korea, platinum-(approximately 71%) or taxane-based (approximately 26%) therapy was selected as the first-line treatment regimen [56]. A report from the Mayo Clinic in the United States indicated that, although the number of cases was limited, various regimens were used, including carboplatin plus paclitaxel, irinotecan, and FP [57].

Although the efficacy of chemotherapy varies depending on the regimen and generally allows for some control of the disease, challenges remain in improving long-term prognosis. Regarding the objective response rate (ORR), the response rates for monthly DOC monotherapy, weekly DOC monotherapy, S-1/DOC therapy, and low-dose FP therapy have been reported to be approximately 51.6% to 58%, 35.7%, 78.6%, and 25% to 59%, respectively [9,13,58,59,60].

The median progression-free survival (PFS) for DOC monotherapy was 6.0 to 7.1 months, and the 1-year PFS rate as determined by the Kaplan–Meier method was 37.0 to 75.0% [9,58,59]. The 1-year PFS rate for S-1/DOC therapy was 46.4% [9]. The median PFS for low-dose FP therapy was 6.3 months, and the 1-year PFS rate was 39.1% [9,13].

The median OS for DOC monotherapy was 16.6 to 26.4 months, and the 1-year OS rate as determined by the Kaplan–Meier method was 77.2% [9,58,59]. The 1-year OS rate for S-1/DOC therapy was 69.2%. The median OS for low-dose FP therapy was 12.0 to 19.4 months, and the 1-year OS rate was 66.1% [9,13,60].

Hematological toxicity is the primary side effect of systemic chemotherapy, particularly in regimens centered on taxane-based agents. High rates of grade 3 or higher neutropenia have been reported with monthly DOC monotherapy and S-1/DOC therapy (approximately 52.7% and 71.4%, respectively). However, adverse events can be managed; in a large-scale Japanese study, the rate of treatment discontinuation due to adverse events was approximately 14% to 28%, and no significant differences were observed between treatment regimens [9].

Currently, in Japan, a phase II, single-arm, physician-initiated clinical trial is underway to evaluate the efficacy of eribulin (a microtubule inhibitor) in patients with EMPD that is not amenable to curative resection [61] (Table 1).

#### 3.3.2. Immune Checkpoint Therapy

Immune checkpoint inhibitors (ICIs), which have demonstrated dramatic efficacy against other types of cancers, are attracting attention as new treatment options for metastatic EMPD [62,63]. Among the anti-programmed cell death-1 (PD-1) antibodies, pembrolizumab has been approved in the United States, Europe, Japan, and other countries worldwide for the treatment of solid tumors with a high tumor mutational burden (TMB) and/or high microsatellite instability. Nivolumab was approved in Japan in 2024 for the treatment of unresectable, advanced, or recurrent epithelial skin malignancies [64]. Therefore, anti-PD-1 antibody therapy is also used to treat EMPD. The NMSC-PD1 trial, conducted in Japan, was a phase II, single-arm, physician-initiated clinical trial designed to evaluate the efficacy and safety of nivolumab in patients with unresectable advanced or recurrent epithelial skin malignancies. The results of this trial showed an ORR of 22.6% and a median duration of response of 21.3 months. However, only four patients with EMPD were enrolled in this trial, and a response was confirmed in only one of these patients [64] (Table 1).

Although clinical data on the efficacy of anti-PD-1 antibodies against EMPD are limited, these agents are expected to be effective based on the several biological and immunological mechanisms. Among patients with EMPD, there are cases with a high TMB. According to an analysis of Japan’s C-CAT database, approximately 21.5% of patients with EMPD are reported to have a high TMB (TMB-high [≥10 mut/Mb]) [63]. Generally, tumors with high TMB tend to respond well to ICIs [65]. On the other hand, regarding MSI, there are reports indicating that, among 101 cases of EMPD, 0% were found to be MSI-high [66]. As for the tumor microenvironment in EMPD, while the PD-L1 expression rate in EMPD cells is low, at approximately 10–14%, it has been reported that the PD-L1 expression rate in the tumor microenvironment, such as in tumor-infiltrating lymphocytes, reaches 71–72%, suggesting the presence of immune evasion mechanisms [62,67,68].

Treatment outcomes with ICIs for advanced EMPD reported to date have ranged from dramatic responses to limited effectiveness. Ishiguro et al. reported a case of invasive EMPD in the groin in which pembrolizumab was administered after confirmation of high TMB (12.07 mut/Mb); all metastatic lesions disappeared 7 months after the start of ICI therapy, resulting in a complete response [69]. Horasaki et al. reported a case with a high TMB (13.0 mut/Mb) that achieved a partial response after switching from chemotherapy to pembrolizumab. However, the response was transient, and the disease progressed 6 months after treatment initiation [65]. In contrast, Narasaki et al. reported three cases in which anti-PD-1 antibodies were administered following disease progression during DOC therapy, resulting in either disease stabilization or further progression; they noted that even in cases with high TMB, sufficient therapeutic efficacy was not achieved [63]. Guercio et al. reported a case in which a patient with metastatic EMPD who was resistant to chemotherapy and targeted molecular therapies received combination therapy with ipilimumab (1 mg/kg) and nivolumab (3 mg/kg) and achieved a sustained partial response over a 7-month period, despite low PD-L1 expression and low TMB [62]. Thus, real-world clinical data on the efficacy of ICIs for EMPD are limited to case reports, and treatment responses vary among reports. Furthermore, findings from the C-CAT database suggest that high TMB status alone is insufficient to reliably predict ICI efficacy [63]. It will be necessary to identify other treatment response biomarkers to select patients who can benefit from ICIs.

#### 3.3.3. Anti-HER2 Antibody Therapy

Although no standard systemic chemotherapy regimen has been established for metastatic EMPD, HER2-targeted therapy is a promising option because its immunohistochemical profile in EMPD resembles that of breast cancer, and HER2 overexpression or gene amplification is observed in approximately 30–40% of cases [70,71]. In addition to HER2 overexpression and gene amplification, activating somatic mutations in the erb-b2 receptor tyrosine kinase (ERBB)2 gene represent another clinically significant mechanism of *HER2* activation. Generally referred to as *HER2* mutations, *ERBB2* mutations, or *HER2/neu* mutations, these changes occur independently of gene amplification and result in the sustained activation of HER2 signaling. Reported mutations are located within the kinase domain (e.g., D769Y, D769H, V842I, I767M, R678Q, S310F) or the extracellular domain and promote ligand-independent receptor activation [71,72,73,74,75]. These mutations are attracting attention as important prognostic biomarkers for HER2-targeted therapies, particularly antibody–drug conjugates (ADCs) and tyrosine kinase inhibitors. In the United States, Europe, and Japan, trastuzumab deruxtecan (brand name: Enhertu) is indicated for HER2-positive advanced or recurrent solid tumors across various cancer types (limited to cases where standard treatment is not feasible). There are at least three distinct biological and laboratory concepts associated with “HER2-positive.” Specifically, these are HER2 protein overexpression detected by immunohistochemistry (IHC), *ERBB2* gene amplification detected by in situ hybridization/fluorescence in situ hybridization or Next-Generation Sequencing (NGS), and *ERBB2* activating mutations detected by NGS. While these concepts may correlate, they are not synonymous, and the specific concept adopted for this tissue-agnostic indication varies by country. In Europe and the United States, the definition of HER2 includes HER2 overexpression in tumor tissue as determined by IHC 3+; in Japan, the definition includes both HER2 overexpression in tumor tissue as determined by IHC 3+ and *HER2* (*ERBB2*) gene amplification detected in plasma cfDNA. However, as of June 2026, the only approved diagnostic test available in Japan for solid tumor indications is the Guardant360 CDx cancer gene panel, which detects *ERBB2* copy number abnormalities in blood; there are no approved diagnostic tests that assess IHC 3+ across cancer types. Under these circumstances, it is suggested that the drug cannot be administered for the relevant solid tumor indications based solely on IHC 3+ results. Several case reports and retrospective studies have reported the efficacy of HER2-targeted therapies such as trastuzumab and trastuzumab emtansine for the treatment of EMPD. Trastuzumab has been reported to be effective in combination with paclitaxel [71,76]. Incognito et al. reported that trastuzumab monotherapy may be a viable option in cases in which cytotoxic chemotherapy is difficult to administer because of severe side effects [77]. In Japan, a prospective phase II single-arm trial (EMPD-HER2DOC trial) was conducted to evaluate the efficacy and safety of trastuzumab and DOC combination therapy in HER2-positive advanced EMPD [70] (Table 1). This trial enrolled 13 patients aged 20 years or older with an performance status of 0 or 1; a left ventricular ejection fraction of 50% or higher; and adequate bone marrow, liver, and kidney function. The criteria for HER2-positive status in this study were defined as HER2 immunohistochemistry 3+ or 2+ with confirmation of *HER2* gene amplification by in situ hybridization, based on the HER2 testing algorithm for breast cancer of the American Society of Clinical Oncology/College of American Pathologists guidelines [70]. The treatment regimen consisted of trastuzumab (8 mg/kg as the first dose, followed by 6 mg/kg) and DOC (75 mg/m^2^) administered every 3 weeks. In this phase II trial, extremely favorable results were obtained, with an ORR of 76.9% and a disease control rate of 100%. The complete response rate was 38.5%. The median PFS was 9.3 months (95% confidence interval: 5.8–30.4 months), the median overall survival was not reached during the follow-up period (median 27.9 months), and the 3-year survival rate was estimated to be 53.7%. Adverse events included hematological toxicity in the form of neutropenia, which was observed in all cases, with 92.3% of cases being grade 4. This necessitated the use of granulocyte-colony stimulating factor agents and adjustment of the initial DOC dose. Non-hematological toxicities included low albumin levels (84.6%), mucocutaneous infections (84.6%), alopecia (76.9%), and peripheral neuropathy (46.2%). A rare adverse event was treatment discontinuation due to grade 1 macular edema. The combination of DOC and trastuzumab demonstrated favorable clinical effects and acceptable tolerability in phase 2 trials, suggesting that it may be an effective treatment option for HER2-positive metastatic EMPD [70].

As a next-generation treatment targeting HER2, ADCs, which combine HER2 with anticancer drugs, are attracting attention. In Japan, a phase II single-arm, physician-initiated clinical trial is currently underway to evaluate the efficacy and safety of trastuzumab emtansine in patients with HER2-positive advanced EMPD (TEMENOS2 trial, jRCT2031210436). For the purposes of this study, HER2-positive status was defined as HER2 immunohistochemistry grade 3+ or confirmed *HER2* gene amplification by in situ hybridization in accordance with the breast cancer HER2 testing algorithms of the American Society of Clinical Oncology and the College of American Pathologists. Additionally, one clinical trial in China has been registered at ClinicalTrials.gov. A prospective, phase 2, multicenter, single-arm clinical trial (NCT06791070) is currently underway to evaluate the efficacy and safety of combination therapy with dicitabine, bedotin, and triparimab (an anti-PD-1 antibody) in patients with advanced HER2-positive EMPD (Table 1). In these trials, the criteria for HER2-positive status were defined as cases confirmed to be HER2-positive by pathology (i.e., those with a HER2 score of 1+ or higher on immunohistochemical testing).

#### 3.3.4. Hormone Therapy

Androgen receptor positivity ranges from 53.6% to 100%, estrogen receptor positivity ranges from 0% to 19.44%, and co-expression of both receptors is observed in 16.67% of EMPD cases [2,16,78,79,80,81]. Androgen receptor expression was reported to be higher in invasive EMPD than in intra-epithelial EMPD. This suggests that androgen receptor signaling may be involved in the progression of EMPD, implying that inhibition of androgen receptor signaling could be an effective treatment for EMPD [78,79,80]. Although this is a case report, there have been reports of cases in which combined androgen deprivation therapy using bicalutamide and leuprolide acetate (a luteinizing hormone-releasing hormone agonist), both of which are used to treat prostate cancer, resulted in an improvement in multiple bone metastases associated with EMPD [82].

### 3.4. Future Directions and Perspectives

Based on the current biological insights into EMPD, promising future treatment strategies can be broadly categorized into four main areas. These are the HER2 pathway, new targets for ADCs, immunotherapy, and new molecules involved in cell proliferation and invasion.

As discussed in Section 3.3.3. there are high expectations for HER2-targeted therapy. HER2-centered treatment strategies, particularly ADCs that combine HER2 with anticancer drugs, show promise. According to a retrospective observational study, disitamab vedotin demonstrated a response rate of 73% even in patients with HER2-low or higher expression, suggesting that the use of ADCs may not be limited to HER2-high cases [83]. Watanabe et al. reported that HER2 overexpression was observed in 30% of advanced EMPD cases, whereas oncogenic alterations, including *ERBB2* mutations and amplifications, were observed in 63% of cases analyzed using next-generation sequencing. This suggests that protein expression and gene activation do not necessarily correlate [72].

TROP2 and NECTIN4 are promising targets for ADCs. A Japanese multicenter study reported high TROP2 expression in both primary and metastatic EMPD lesions [84]. In vitro, *TROP2* knockdown resulted in reduced cell viability and motility, and sacituzumab govitecan, an ADC targeting TROP2, inhibited cell proliferation [84]. It has also been reported that NECTIN4 is highly expressed in EMPD and is associated with increased tumor thickness, advanced disease stage, and reduced disease-specific survival. In vitro, knockdown of *NECTIN4* resulted in reduced cell proliferation and migration, and enfortumab–monomehtyl auristatin E (the cytotoxic payload), an ADC targeting NECTIN4, reduced cell viability [85,86]. These results suggest that ADCs targeting TROP2 and NECTIN4 are promising treatment options for unresectable EMPD. However, because both proteins are also expressed in normal skin and appendages, it is important to note that there may be a high risk of skin toxicity.

The future of immunotherapy lies in appropriate patient selection, which is key to realizing the therapeutic benefits of ICIs. In EMPD, the prevalence of microsatellite instability-high was low [64,66], and PD-L1 was positive only in a subset of cases [87]. According to an analysis of Japan’s C-CAT database, approximately 21.5% of patients with EMPD are reported to have high TMB (≥10 mut/Mb [63]); however, cases have been reported where anti-PD-1 antibodies were ineffective even in patients with high TMB, suggesting that TMB alone is not a sufficient biomarker. Therefore, it is necessary to identify patients who are suitable candidates for ICIs using a combination of biomarkers, such as TMB-high status, high infiltration of CD8^+^-T cells, and PD-1/PD-L1 expression. Further research is needed to identify more appropriate biomarkers. Furthermore, while combination therapy with anti-PD-1 antibodies and molecularly targeted drugs is a treatment option for other types of cancer, a phase 2 single-arm clinical trial targeting advanced EMPD found no response to the combination of camrelizumab (an anti-PD-1 antibody) and rivoceranib (a vascular endothelial growth factor receptor inhibitor) [87] (Table 1). In this trial, 11 patients received the investigational treatment. The primary endpoint was the ORR, with a threshold of 10% and an expected value of 35%. The results showed an ORR of 0%, and the statistical criteria were not met. The median PFS, a secondary endpoint, was 2.4 months (95% confidence interval: 2.1–4.4 months). Regarding adverse events, Grade 3 adverse events were observed in 6 patients [87]. Therefore, identifying a therapeutic agent that is most likely to yield synergistic effects when combined with anti-PD-1 antibodies is a key challenge.

Several other potential therapeutic targets have been identified. Ito et al. reported an association between the transcription factor FOXM1 and EMPD and noted that FOXM1 is expressed in both primary and metastatic lesions of EMPD [88]. They also reported that patients in whom FOXM1 was expressed in more than 10% of the tumor cells had a significantly shorter disease-specific survival than other patients. In vitro studies have shown that the knockdown of *FOXM1* results in reduced cell viability, migration, and invasion, suggesting that FOXM1 may be a promising therapeutic target [88]. Furthermore, Takeuchi et al. reported that targeted sequencing detected mutations in *FOXA1* and *PIK3CA* in samples from patients with EMPD and that immunostaining revealed strong expression of FOXA1 in tumor cell nuclei [89]. The authors explained that the upregulation of FOXA1, a transcription factor for the estrogen receptor, suggests that treatments for hormone-dependent cancers may also be effective against EMPD. With regard to PIK3CA, Huang et al. have reported cases in which patients with EMPD harboring *PIK3CA* gene mutations and distant metastases responded to treatment with PIK3CA inhibitors as a second-line therapy [90].

Translational research that bridges the gap between in vitro studies and clinical trials is of great importance.

## 4. Conclusions

**Figure 2:**
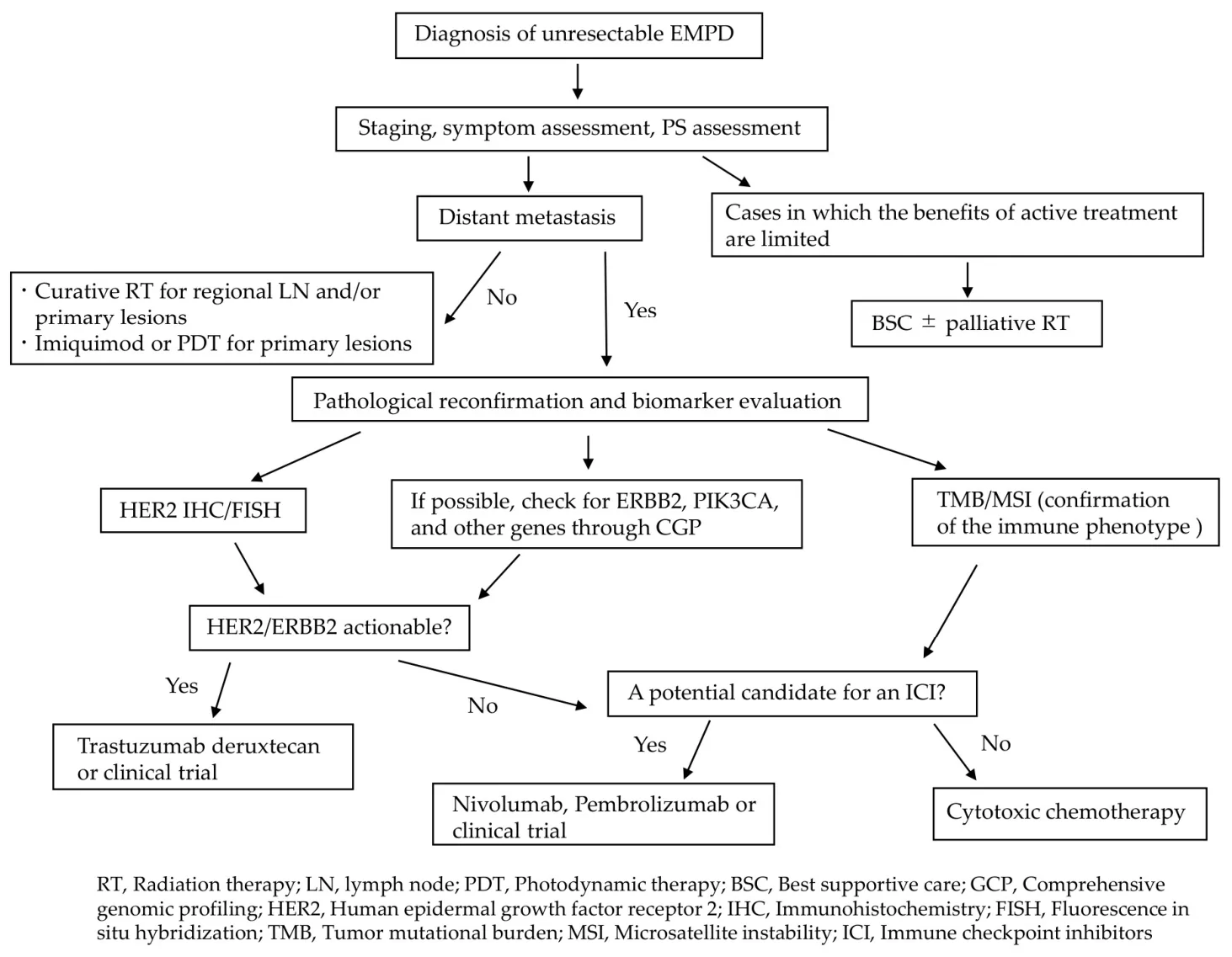
Proposed treatment strategy flowchart for unresectable EMPD.

EMPD remains a challenging malignancy because of its rarity, clinical heterogeneity, and limited evidence. Although most patients present with a localized disease that can be effectively managed with surgery, a substantial proportion eventually develop invasive disease, regional lymph node metastases, or distant metastases, all of which are associated with significantly worse outcomes. Therefore, treatment strategies should be individualized according to the disease extent, patient condition, and underlying tumor biology. Figure 2 shows a proposed flowchart of treatment strategies for unresectable EMPD.

For localized diseases, surgical excision remains the gold standard. Advances in margin assessment techniques, including MMS, CCPDMA, and mapping biopsy-guided surgery, have improved local control while minimizing unnecessary tissue sacrifices. For patients who are unsuitable candidates for surgery, radiotherapy is the most established alternative, whereas topical imiquimod and PDT may provide additional options in carefully selected cases.

For advanced EMPD, systemic treatment has historically relied on cytotoxic chemotherapy; however, long-term disease control remains unsatisfactory. Recent molecular studies have fundamentally improved our understanding of EMPD biology and identified multiple actionable therapeutic targets. Advances in molecular profiling have revealed several actionable targets, including HER2, androgen receptor signaling pathways, immune checkpoint pathways, TROP2, NECTIN4, FOXM1, and PIK3CA. Among these, HER2-targeted therapy has demonstrated the most compelling clinical evidence to date and is likely to become the cornerstone of treatment for HER2-positive advanced disease. In addition, the development of ADCs and biomarker-driven immunotherapy represents a promising direction for future therapeutic innovations.

Finally, the main findings of this narrative review are summarized in Table 2. As our understanding of EMPD biology continues to expand, treatment strategies are expected to shift toward personalized medicine. The integration of molecular diagnostics, predictive biomarkers, and targeted therapeutics will enable personalized treatment selection and may substantially improve the outcomes for patients with advanced disease. Given the rarity of EMPD, international collaborative research, multicenter prospective studies, and translational investigations linking laboratory discoveries to clinical applications are crucial to establish evidence-based treatment strategies and realize the full potential of precision medicine for this disease.

## Figures and Tables

**Table 1:** Clinical trials related to extramammary Paget disease.

Study/ Registration Number	Phase	Target Population (Number of Patients)	Administered Drugs	Primary Endpoint	Results
EMPHASIS/ jRCT2011220022	Single-arm phase II	Unresectable EMPD (18)	Eribulin	ORR	As of May 2026, the results had not been published in a scientific journal.
NMSC-PD1/ jRCT2031190048	Single-arm phase II	Epithelial skin malignancies not amenable to curative resection or that have recurred (31)	nivolumab 480 mg Q4W	BICR-ORR	ORR 22.6% EMPD showed a response in 1 out of 4 cases
EMPD-HER2DOC/ jRCTs031180073	Single-arm phase II	HER2-positive metastatic EMPD, IHC 3+ or IHC 2+/ISH+ (13)	Trastuzumab + docetaxel	ORR and safety	ORR 76.9%, median PFS 9.3 months, OS not yet reached
TEMENOS2 trial/jRCT2031210436	Single-arm phase II	HER2-positive metastatic EMPD, IHC 3+ or ISH+ (12)	trastuzumab emtansine	BICR-ORR	As of May 2026, the results had not been published in a scientific journal.
2400086153	Single-arm phase II	Advanced EMPD (12)	camrelizumab + rivoceranib	ORR	ORR 0%, median PFS 2.4 months
NCT06791070	Single-arm phase II	Advanced HER2-positive EMPD, IHC ≧ 1+	disitamab vedotin + toripalimab	ORR	As of May 2026, the results had not been published in a scientific journal.

BICR, blinded independent central review; EMPD, extramammary Paget disease; IHC, immunohistochemistry; ISH, in situ hybridization; ORR, objective response rate; PFS, progression-free survival; OS, overall survival.

**Table 2:** Key findings of our narrative review.

Category	Key Findings	Clinical Considerations
Primary lesion (resectable disease)	Surgical excision remains the standard treatment. Margin-controlled surgical techniques, including MMS, CCPDMA, and excision guided by mapping biopsy, contribute to improved local disease control.	Margin-controlled surgery is recommended to achieve accurate assessment of tumor boundaries and minimize local recurrence.
Primary lesion (unresectable disease)	Radiation therapy, topical imiquimod, and PDT represent effective alternative treatment options.	These modalities are particularly useful for cases who cannot undergo surgery; however, recurrence rates remain relatively high.
Regional lymph node metastasis	Management strategies include SLNB, lymph node dissection, and postoperative radiotherapy, selected according to individual clinical circumstances.	Optimal indications for SLNB remain controversial, and recommendations differ among current clinical guidelines.
Advanced or metastatic disease: Chemotherapy	Cytotoxic chemotherapy, primarily taxane-based regimens and fluoropyrimidine/platinum combinations, demonstrates modest clinical activity.	Although antitumor responses have been reported, their durability is generally limited, and no globally accepted standard regimen has been established.
Advanced or metastatic disease: HER2-targeted therapy	HER2 overexpression or gene amplification is observed in approximately 30–40% of patients. HER2-targeted therapies, particularly trastuzumab-based regimens, have demonstrated substantial clinical efficacy. ADCs, including trastuzumab deruxtecan, have also shown promising therapeutic potential.	HER2 currently represents the molecular target supported by the strongest clinical evidence in EMPD.
Advanced or metastatic disease: ICIs	ICIs have demonstrated clinical activity in selected patients, particularly those with high tumor mutational burden (TMB-high).	Predictive biomarkers have not yet been fully established, highlighting the importance of appropriate patient selection.
Emerging molecular targets	Novel therapeutic targets, including, AR, TROP2, NECTIN4, FOXM1, and the PIK3CA signaling pathway, have emerged as promising candidates for future targeted therapies.	Further validation through prospective clinical trials is required.
Future perspectives	Clinical management is progressively shifting toward biomarker-driven precision medicine.	HER2-targeted therapy, ADCs, and biomarker-guided immunotherapy are expected to become central treatment strategies. International collaborative studies and prospective clinical trials will be essential to establish evidence-based therapeutic standards.

MMS, Mohs micrographic surgery; CCPDMA, complete circumferential peripheral and deep margin assessment; PDT, photodynamic therapy; SLNB, sentinel lymph node biopsy; HER2, Human epidermal growth factor receptor 2; ADC, Antibody–drug conjugates; EMPD, extramammary Paget disease; ICI, Immune checkpoint inhibitors; AR, androgen receptor.

## Data Availability

No new data were created or analyzed in this study. Data sharing is not applicable to this article.

## Abbreviations

The following abbreviations are used in this manuscript:

ADCs	Antibody–drug conjugates
CCPDMA	Complete circumferential peripheral and deep margin assessment
CK	Cytokeratin
EMPD	Extramammary Paget disease
ERBB	Erb-b2 receptor tyrosine kinase
FP	5-FU/cisplatin
GCDFP	Gross cystic disease fluid protein
HER2	Human epidermal growth factor receptor 2
HpD	Hematoporphyrin derivative
ICIs	Immune checkpoint inhibitors
IHC	Immunohistochemistry
LRR	Local recurrence rate
MMS	Mohs micrographic surgery
NGS	Next-Generation Sequencing
ORR	Objective response rate
OS	Overall survival
PDT	Photodynamic therapy
PD-1	Programmed cell death-1
PFS	Progression-free survival
RCTs	Randomized controlled trials
SLNB	Sentinel lymph node biopsy
TMB	Tumor mutational burden
5-ALA	5-aminolevulinic acid
5-FU	5-fluorouracil

## References

[1] Desai S., McCormick E., Nelson K., Friedman A. (2023). EXTRA, EXTRA, Treatment Approaches for EXTRAmammary Paget Disease. J. Drugs Dermatol..

[2] Ishizuki S., Nakamura Y. (2021). Extramammary Paget’s Disease: Diagnosis, Pathogenesis, and Treatment with Focus on Recent Developments. Curr. Oncol..

[3] Matsushita S., Kajihara I., Tsutsui K., Hirai I., Maekawa T., Maeda T., Okuma K., Kimura T., Komori T., Sato S. (2025). Japanese Dermatological Association Guidelines: Clinical Questions of Guidelines for Extramammary Paget Disease 2025. J. Dermatol..

[4] Kibbi N., Owen J.L., Worley B., Wang J.X., Harikumar V., Downing M.B., Aasi S.Z., Aung P.P., Barker C.A., Bolotin D. (2022). Evidence-Based Clinical Practice Guidelines for Extramammary Paget Disease. JAMA Oncol..

[5] Lloyd J., Flanagan A.M. (2000). Mammary and extramammary Paget’s disease. J. Clin. Pathol..

[6] Weng S., Zhu N., Li D., Chen Y., Tan Y., Chen J., Yuan Y. (2020). Clinical Characteristics, Treatment, and Prognostic Factors of Patients with Primary Extramammary Paget’s Disease (EMPD): A Retrospective Analysis of 44 Patients from a Single Center and an Analysis of Data from the Surveillance, Epidemiology, and End Results (SEER) Database. Front. Oncol..

[7] Nowak M.A., Guerriere-Kovach P., Pathan A., Campbell T.E., Deppisch L.M. (1998). Perianal Paget’s disease: Distinguishing primary and secondary lesions using immunohistochemical studies including gross cystic disease fluid protein-15 and cytokeratin 20 expression. Arch. Pathol. Lab. Med..

[8] Hashimoto H., Ito T. (2022). Current Management and Treatment of Extramammary Paget’s Disease. Curr. Treat. Options Oncol..

[9] Miyashita A., Fukushima S., Yoshino K., Kato H., Yamazaki N., Kawashima S., Yamamoto Y., Nakamura Y., Kiniwa Y., Ishizuki S. (2026). Real-World Efficacy of Chemotherapy in Patients with Advanced Unresectable Extramammary Paget Disease: A Retrospective Multicenter Study of 138 Japanese Patients. J. Dermatol..

[10] Fujisawa Y., Otsuka F. (2013). Epidemiologic survey of malignant skin tumor-international comparison between Japan and foreign countries. Nihon Rinsho.

[11] Ohara K., Fujisawa Y., Yoshino K., Kiyohara Y., Kadono T., Murata Y., Uhara H., Hatta N., Uchi H., Matsushita S. (2016). A proposal for a TNM staging system for extramammary Paget disease: Retrospective analysis of 301 patients with invasive primary tumors. J. Dermatol. Sci..

[12] Ogata D., Namikawa K., Nakano E., Fujimori M., Uchitomi Y., Higashi T., Yamazaki N., Kawai A. (2023). Epidemiology of skin cancer based on Japan’s National Cancer Registry 2016–2017. Cancer Sci..

[13] Kato H., Watanabe S., Kariya K., Nakamura M., Morita A. (2018). Efficacy of low-dose 5-fluorouracil/cisplatin therapy for invasive extramammary Paget’s disease. J. Dermatol..

[14] Ohata K., Onishi Y., Kawabata Y. (1993). Diagnosis and treatment of extramammary Paget’s disease. Skin Cancer.

[15] Hatta N., Yamada M., Hirano T., Fujimoto A., Morita R. (2008). Extramammary Paget’s disease: Treatment, prognostic factors and outcome in 76 patients. Br. J. Dermatol..

[16] Perez J.C., Salgado A.C., Perez-Mies B., Rullan J.A.D., Ajuria-Illarramendi O., Alia E.M.G., Serrano Domingo J.J. (2023). Extramammary Paget Disease: A Therapeutic Challenge, for a Rare Entity. Curr. Oncol. Rep..

[17] Wollina U., Goldman A., Bieneck A., Abdel-Naser M.B., Petersen S. (2018). Surgical Treatment for Extramammary Paget’s Disease. Curr. Treat. Options Oncol..

[18] Yang J., Yan J., Luo H., Li L., Jiang L., Chen X., Wu L., Chai J., Xue H., Gan L. (2025). Narrow-Margin Treatment with Modified Mohs Microsurgery for Extramammary Paget’s Disease: A Retrospective Clinical Analysis. Clin. Cosmet. Investig. Dermatol..

[19] Song C., Li J.H., Luo X.Y., Zhou P., Yang L.F., Peng J.Z. (2023). The clinical effect of modified slow Mohs circular skin biopsy on penoscrotal extramammary Paget’s disease. Asian J. Surg..

[20] Park S.O., Ha J.H., Hong K.Y., Chang H. (2017). Usefulness of Mapping Biopsy in the Treatment of Penoscrotal Extramammary Paget’s Disease. Ann. Surg. Oncol..

[21] Jung J.H., Kwak C., Kim H.H., Ku J.H. (2013). Extramammary Paget Disease of External Genitalia: Surgical Excision and Follow-up Experiences with 19 Patients. Korean J. Urol..

[22] Kato T., Fujimoto N., Fujii N., Tanaka T. (2013). Mapping biopsy with punch biopsies to determine surgical margin in extramammary Paget’s disease. J. Dermatol..

[23] Ikari S., Otsuka M., Yamamoto T. (2021). Clinical analysis of 65 patients with extramammary Paget’s disease at Department of Dermatology, Fukushima Medical University between 2006 and 2017. Jpn. J. Clin. Dermatol..

[24] Hata M., Koike I., Wada H., Miyagi E., Kasuya T., Kaizu H., Mukai Y., Inoue T. (2015). Postoperative radiation therapy for extramammary Paget’s disease. Br. J. Dermatol..

[25] Itonaga T., Nakayama H., Okubo M., Mikami R., Nogi S., Tajima Y., Sugahara S., Tokuuye K. (2014). Radiotherapy in patients with extramammary Paget’s disease—Our own experience and review of the literature. Oncol. Res. Treat..

[26] Tagliaferri L., Casa C., Macchia G., Pesce A., Garganese G., Gui B., Perotti G., Gentileschi S., Inzani F., Autorino R. (2018). The Role of Radiotherapy in Extramammary Paget Disease: A Systematic Review. Int. J. Gynecol. Cancer.

[27] Zhang M., Gao X., Gao T., Li H., Sheng X., Su M., Jia C. (2026). A retrospective study on Mohs micrographic surgery combined with adjuvant radiotherapy in the treatment of extramammary Paget’s disease: Analysis of 87 patients. Postgrad. Med. J..

[28] Min Z., Xianshu G., Tianjing G., Hang L., Xueqing S., Mengmeng S., Chenghao J. (2026). Efficacy of Radiation Therapy in 106 Patients with Extramammary Paget Disease: A Retrospective Study. Adv. Radiat. Oncol..

[29] Cowan R.A., Black D.R., Hoang L.N., Park K.J., Soslow R.A., Backes F.J., Gardner G.J., Abu-Rustum N.R., Leitao M.M., Eisenhauer E.L. (2016). A pilot study of topical imiquimod therapy for the treatment of recurrent extramammary Paget’s disease. Gynecol. Oncol..

[30] Mayo-Martinez F., Moro R., Millan-Esteban D., Rios-Vinuela E., Bautista I.J., Nagore E., Sanmartin O., Llombart B. (2023). Topical Imiquimod in Primary Cutaneous Extramammary Paget’s Disease: A Systematic Review. Cancers.

[31] Liau M.M., Yang S.S., Tan K.B., Aw C.W. (2016). Topical imiquimod in the treatment of extramammary Paget’s disease: A 10 year retrospective analysis in an Asian tertiary centre. Dermatol. Ther..

[32] Borella F., Preti M., Vieira-Baptista P., Perez-Lopez F.R., Bertero L., Gallio N., Micheletti L., Benedetto C. (2022). Vulvar Paget’s disease: Outcomes of 51 patients treated with imiquimod cream. Maturitas.

[33] van der Linden M., van Hees C.L., van Beurden M., Bulten J., van Dorst E.B., Esajas M.D., Meeuwis K.A., Boll D., van Poelgeest M.I., de Hullu J.A. (2022). The Paget Trial: Topical 5% imiquimod cream for noninvasive vulvar Paget disease. Am. J. Obstet. Gynecol..

[34] Sawada M., Kato J., Yamashita T., Yoneta A., Hida T., Horimoto K., Sato S., Uhara H. (2018). Imiquimod 5% cream as a therapeutic option for extramammary Paget’s disease. J. Dermatol..

[35] Ren F., Zhao S., Yang C., Liu J., Shang Q., Feng K., Kang X., Zhang R., Wang X., Wang X. (2023). Applications of photodynamic therapy in extramammary Paget’s disease. Am. J. Cancer Res..

[36] Shim P.J., Zeitouni N.C. (2020). Photodynamic therapy for extramammary Paget’s disease: A systematic review of the literature. Photodiagnosis Photodyn. Ther..

[37] Chen F., Sun L., Zeng R., Chen X., Li L., Cai X. (2025). Photodynamic therapy for extramammary paget’s disease: A retrospective analysis of 15 cases. Photodiagnosis Photodyn. Ther..

[38] Wang D., Wang P., Li C., Zhou Z., Zhang L., Zhang G., Wang X. (2022). Efficacy and safety of HpD-PDT for Extramammary Paget’s Disease refractory to conventional therapy: A prospective, open-label and single arm pilot study. Photodiagnosis Photodyn. Ther..

[39] Li X., Zhao C., Kou H., Zhu F., Yang Y., Lu Y. (2022). PDD-guided tumor excision combined with photodynamic therapy in patients with extramammary Paget’s disease. Photodiagnosis Photodyn. Ther..

[40] Hata M., Koike I., Wada H., Miyagi E., Odagiri K., Minagawa Y., Kasuya T., Kaizu H., Inoue T. (2012). Definitive radiation therapy for extramammary Paget’s disease. Anticancer Res..

[41] Tackenberg S., Gehrig A., Dummer R., Navarini A.A. (2015). External beam radiotherapy of extramammary Paget disease. Cutis.

[42] Tanimura H., Tsuda M., Shijimaya T., Nagano N., Nakamaru S., Makimura K., Kiyohara T. (2021). Six Cases of Extramamammary Paget’s Disease Treated with Radiotherapy. Hifunokagaku.

[43] Hata M., Koike I., Wada H., Miyagi E., Kasuya T., Kaizu H., Matsui T., Mukai Y., Ito E., Inoue T. (2014). Radiation therapy for extramammary Paget’s disease: Treatment outcomes and prognostic factors. Ann. Oncol..

[44] Niwa M., Tomita N., Ishiyama H., Kaneko H., Oshima Y., Takano H., Matsuo M., Kuno M., Miyakawa A., Otsuka S. (2025). Clinical Outcomes and Prognostic Factors for Extramammary Paget’s Disease Treated with Radiation Therapy: A Multi-Institutional Observational Study. Cancers.

[45] Maeda T., Nagai K., Uehara J., Toyoshima R., Nakagawa T., Yoshino K. (2023). Sentinel lymph node biopsy in extramammary Paget disease: A 13-year institutional experience. J. Dermatol..

[46] Faisel L., Swanson A., Sheridan C., Walker T., Carr D.R., Shahwan K.T. (2023). The role of sentinel lymph node biopsy in extramammary paget disease: A systematic review. Arch. Dermatol. Res..

[47] Fujisawa Y., Yoshino K., Kiyohara Y., Kadono T., Murata Y., Uhara H., Hatta N., Uchi H., Matsushita S., Takenouchi T. (2015). The role of sentinel lymph node biopsy in the management of invasive extramammary Paget’s disease: Multi-center, retrospective study of 151 patients. J. Dermatol. Sci..

[48] Ogata D., Kiyohara Y., Yoshikawa S., Tsuchida T. (2016). Usefulness of sentinel lymph node biopsy for prognostic prediction in extramammary Paget’s disease. Eur. J. Dermatol..

[49] Kurooka S., Namikawa K., Tsutsumida A., Tanaka R., Kato J., Yamazaki N. (2012). Our Department’s Experience in Treating Extramammary Paget’s Disease over the Past 10 Years. Jpn. J. Dermatol..

[50] Yoshino K., Yamazaki N., Yamamoto A., Namikawa K., Yoshida H. (2006). Lymph Node Metastasis and Sentinel Lymph Node Biopsy in the Extramamary Paget’s Disease. Jpn. J. Dermatol..

[51] Matsushita S., Hatanaka M., Katsue H., Kawai K., Kanekura T. (2013). Possible effectiveness of lymph node dissection in patients with extramammary Paget’s disease. J. Dermatol..

[52] Ito T., Kaku Y., Nagae K., Nakano-Nakamura M., Nakahara T., Oda Y., Hagihara A., Furue M., Uchi H. (2015). Tumor thickness as a prognostic factor in extramammary Paget’s disease. J. Dermatol..

[53] Tsutsui K., Takahashi A., Muto Y., Mizuta H., Jinnai S., Nakama K., Ogata D., Namikawa K., Yamazaki N. (2020). Outcomes of lymph node dissection in the treatment of extramammary Paget’s disease: A single-institution study. J. Dermatol..

[54] Okumura M., Ogata D., Namikawa K., Tsutsui K., Takahashi A., Okuma K., Kashihara T., Igaki H., Akiyama M., Yamazaki N. (2022). Postoperative radiation therapy improves prognoses in extramammary Paget’s disease presenting with multiple lymph node metastases. J. Dermatol..

[55] Hata M., Koike I., Wada H., Minagawa Y., Kasuya T., Matsui T., Suzuki R., Takano S., Inoue T. (2014). Radiation therapy for lymph node metastasis from extramammary Paget’s disease. J. Eur. Acad. Dermatol. Venereol..

[56] Sohn B.S., Kim J., Kim M., Hong J.Y., Lee J., Park S.E., Kim H., Lee H.J., Kang E.J., Lee S.I. (2023). Treatment outcomes of advanced/metastatic extramammary Paget’s disease in Korean patients: KCSG-RC20-06. Cancer Med..

[57] Padrnos L., Karlin N., Halfdanarson T.R. (2016). Mayo Clinic Cancer Center Experience of Metastatic Extramammary Paget Disease 1998–2012. Rare Tumors.

[58] Nakamura Y., Tanese K., Hirai I., Fukuda K., Kawakami Y., Amagai M., Funakoshi T. (2020). Weekly docetaxel monotherapy for metastatic extramammary Paget’s disease: Retrospective single-institute analysis. J. Dermatol..

[59] Yoshino K., Fujisawa Y., Kiyohara Y., Kadono T., Murata Y., Uhara H., Hatta N., Uchi H., Matsushita S., Takenouchi T. (2016). Usefulness of docetaxel as first-line chemotherapy for metastatic extramammary Paget’s disease. J. Dermatol..

[60] Tokuda Y., Arakura F., Uhara H. (2015). Combination chemotherapy of low-dose 5-fluorouracil and cisplatin for advanced extramammary Paget’s disease. Int. J. Clin. Oncol..

[61] Maeda T., Yanagi T., Tokuchi K., Funakoshi T., Horie N., Isoe T., Ito Y.M., Sato N., Ujiie H. (2024). Eribulin for patients with metastatic extramammary Paget disease: Study protocol for a single-arm phase II trial. Exp. Dermatol..

[62] Guercio B.J., Iyer G., Kidwai W.Z., Lacouture M.E., Ghafoor S., Rossi A.M., Assis D.N., Chen Y.B., Busam K.J., Janjigian Y.Y. (2021). Treatment of Metastatic Extramammary Paget Disease with Combination Ipilimumab and Nivolumab: A Case Report. Case Rep. Oncol..

[63] Narasaki M., Kato J., Sato S., Hida T., Horimoto K., Matsui Y., Shigyo N., Uhara H. (2025). Extramammary Paget’s disease treated with of anti-programmed cell death protein 1 therapy after docetaxel therapy failure. J. Dermatol..

[64] Nakamura Y., Yamazaki N., Namikawa K., Uchi H., Mori S., Ogawa-Momohara M., Fujimura T., Takenouchi T., Kurimoto M., Yamamoto Y. (2026). Nivolumab for unresectable cutaneous epithelial malignancies: An open-label, single-arm, multi-centre, phase II trial (NMSC-PD1). BMC Cancer.

[65] Horisaki K., Taki T., Mori S., Okumura M., Akiyama M. (2024). A Case of Advanced Extramammary Paget’s Disease with a High Tumor Mutation Burden That Showed Partial Lymph Node Regression with Pembrolizumab. Cureus.

[66] Kashiwada-Nakamura K., Myangat T.M., Kajihara I., Kusaba Y., Tanaka K., Sakamoto R., Maeda-Otsuka S., Yamada-Kanazawa S., Sawamura S., Kanemaru H. (2021). Absence of microsatellite instability in extramammary Paget’s disease. Skin Health Dis..

[67] Pourmaleki M., Young J.H., Socci N.D., Chiang S., Edelweiss M., Li Y., Zhang M., Roshal L., Chi D.S., Busam K.J. (2019). Extramammary Paget disease shows differential expression of B7 family members B7-H3, B7-H4, PD-L1, PD-L2 and cancer/testis antigens NY-ESO-1 and MAGE-A. Oncotarget.

[68] Mauzo S.H., Tetzlaff M.T., Milton D.R., Siroy A.E., Nagarajan P., Torres-Cabala C.A., Ivan D., Curry J.L., Hudgens C.W., Wargo J.A. (2019). Expression of PD-1 and PD-L1 in Extramammary Paget Disease: Implications for Immune-Targeted Therapy. Cancers.

[69] Ishiguro A., Mae K., Irisawa R., Hirai H., Harada K. (2025). A Case of Umbilical Invasive Extramammary Paget Disease with a High Tumor Mutational Burden Successfully Treated with Pembrolizumab. Cureus.

[70] Hirai I., Tanese K., Nakamura Y., Fukuda K., Ouchi T., Hayashida T., Kameyama K., Abe T., Amagai M., Funakoshi T. (2024). Phase II clinical trial of docetaxel and trastuzumab for HER2-positive advanced extramammary Paget’s disease (EMPD-HER2DOC). Oncologist.

[71] Pomati G., Corrado G., Palluzzi E., Manna G., Fragomeni S.M., Federico A., Lanzetta G., Scambia G., Garganese G. (2025). The role of HER2 pathway in vulvar paget’s disease. Crit. Rev. Oncol. Hematol..

[72] Watanabe S., Haratani K., Nakayama T., Takahama T., Iwasa T., Takeda M., Hayashi H. (2025). Clinical and molecular landscape of metastatic extramammary Paget’s disease. Oncologist.

[73] Omi M., Takeichi T., Okuno Y., Hsu C.K., Wu C.L., Chang Y.H., Mori S., Yamashita Y., Miyazaki A., Taira T. (2025). Prevalence of *FOXA1* and *ERBB2* activating mutations in extramammary Paget’s disease: A retrospective multicenter analysis of 99 cases from Japanese and Taiwanese cohorts. J. Dermatol. Sci..

[74] Kiniwa Y., Yasuda J., Saito S., Saito R., Motoike I.N., Danjoh I., Kinoshita K., Fuse N., Yamamoto M., Okuyama R. (2019). Identification of genetic alterations in extramammary Paget disease using whole exome analysis. J. Dermatol. Sci..

[75] Stasenko M., Jayakumaran G., Cowan R., Broach V., Chi D.S., Rossi A., Hollman T.J., Zehir A., Abu-Rustum N.R., Leitao M.M. (2020). Genomic Alterations as Potential Therapeutic Targets in Extramammary Paget’s Disease of the Vulva. JCO Precis. Oncol..

[76] Sekiguchi N., Kubota S., Noguchi T., Fukushima T., Kobayashi T., Kanda S., Koizumi T., Miyake T., Shirai T., Okuyama R. (2020). Experiences of trastuzumab plus paclitaxel combination therapy in metastatic human epidermal growth factor receptor 2-positive extramammary Paget’s disease: Four cases and a review. J. Dermatol..

[77] Incognito D., Berretta M., Incognito G.G., Gulino F.A., Foti R., Canzonieri V., Gelsomino C., Palumbo M., Picone A. (2025). Trastuzumab monotherapy as maintenance treatment for metastatic HER2+ vulvar Paget disease: Systematic review and case report. Gynecol. Oncol. Rep..

[78] Liegl B., Horn L.C., Moinfar F. (2005). Androgen receptors are frequently expressed in mammary and extramammary Paget’s disease. Mod. Pathol..

[79] Diaz de Leon E., Carcangiu M.L., Prieto V.G., McCue P.A., Burchette J.L., To G., Norris B.A., Kovatich A.J., Sanchez R.L., Krigman H.R. (2000). Extramammary Paget disease is characterized by the consistent lack of estrogen and progesterone receptors but frequently expresses androgen receptor. Am. J. Clin. Pathol..

[80] Azmahani A., Nakamura Y., Ozawa Y., McNamara K.M., Fujimura T., Haga T., Hashimoto A., Aiba S., Sasano H. (2015). Androgen receptor, androgen-producing enzymes and their transcription factors in extramammary Paget disease. Hum. Pathol..

[81] Zhou S., Zhong W., Mai R., Zhang G. (2017). Mammary and Extramammary Paget’s Disease Presented Different Expression Pattern of Steroid Hormone Receptors. BioMed Res. Int..

[82] Yoneyama K., Kamada N., Kinoshita K., Kawashima T., Otani M., Endo H., Shinkai H., Utani A. (2005). Androgen-deprivation regimen for multiple bone metastases of extramammary Paget disease. Br. J. Dermatol..

[83] Zhang S., Zhou J., Zhao X., Gan H., Peng W., Li W., Sun M., Hu J., Yan W. (2025). Antibody-drug conjugate (disitamab vedotin) therapy targeting HER2-low or higher advanced extramammary Paget’s disease. Oncologist.

[84] Ito T., Tanaka Y., Ogata D., Nishida H., Shiomi T., Tanaka R., Kawaguchi A., Miyashita A., Fukushima S., Shojiguchi N. (2025). A multicenter study on TROP2 as a potential targeted therapy for extramammary Paget disease in Japan. Sci. Rep..

[85] Tanaka Y., Ito T., Murata M., Tanegashima K., Kaku-Ito Y., Nakahara T. (2024). NECTIN4-targeted antibody-drug conjugate is a potential therapeutic option for extramammary Paget disease. Exp. Dermatol..

[86] Murata M., Ito T., Tanaka Y., Kaku-Ito Y., Furue M. (2020). NECTIN4 Expression in Extramammary Paget’s Disease: Implication of a New Therapeutic Target. Int. J. Mol. Sci..

[87] Zhang S., Ju M., Wang Q., Zhu D., Liu Z., Ye X., Li W. (2025). Carelizumab and rivoceranib for advanced extramammary Paget’s disease: An investigator-initiated multicenter, single-arm, phase II trial. Oncologist.

[88] Ito T., Tanaka Y., Kaku-Ito Y., Oda Y., Nakahara T. (2024). FOXM1: A new therapeutic target of extramammary Paget disease. Sci. Rep..

[89] Takeichi T., Okuno Y., Matsumoto T., Tsunoda N., Suzuki K., Tanahashi K., Kono M., Kikumori T., Muro Y., Akiyama M. (2020). Frequent *FOXA1*-Activating Mutations in Extramammary Paget’s Disease. Cancers.

[90] Huang Y., Dong S., Ren J., Li J., Wu C., Chen H., Liu Y., Yuan J., Huang J., Xiao L. (2025). PIK3CA inhibitor treatment for metastatic scrotal extramammary Paget’s disease: A case report and literature review. AME Case Rep..

